# Gene regulation by histone-modifying enzymes under hypoxic conditions: a focus on histone methylation and acetylation

**DOI:** 10.1038/s12276-022-00812-1

**Published:** 2022-07-22

**Authors:** Junil Kim, Hyerim Lee, Sun-Ju Yi, Kyunghwan Kim

**Affiliations:** 1 Department of Biological Sciences and Biotechnology, Chungbuk National University, Cheongju, Chungbuk 28644 Republic of Korea; 2 Center for Ecology and Environmental Toxicology, Chungbuk National University, Cheongju, Chungbuk 28644 Republic of Korea

**Keywords:** Transcriptional regulatory elements, Epigenomics

## Abstract

Oxygen, which is necessary for sustaining energy metabolism, is consumed in many biochemical reactions in eukaryotes. When the oxygen supply is insufficient for maintaining multiple homeostatic states at the cellular level, cells are subjected to hypoxic stress. Hypoxia induces adaptive cellular responses mainly through hypoxia-inducible factors (HIFs), which are stabilized and modulate the transcription of various hypoxia-related genes. In addition, many epigenetic regulators, such as DNA methylation, histone modification, histone variants, and adenosine triphosphate-dependent chromatin remodeling factors, play key roles in gene expression. In particular, hypoxic stress influences the activity and gene expression of histone-modifying enzymes, which controls the posttranslational modification of HIFs and histones. This review covers how histone methylation and histone acetylation enzymes modify histone and nonhistone proteins under hypoxic conditions and surveys the impact of epigenetic modifications on gene expression. In addition, future directions in this area are discussed.

## Hypoxia: revealing effects on gene activation

New sequencing technologies are revealing how cells respond to hypoxia, insufficient oxygen, by managing gene activation. In multicellular organisms, gene activation is managed by how tightly a section of DNA is wound around proteins called histones; genes in tightly packed regions are inaccessible and inactive, whereas those in looser regions can be activated. Kyunghwan Kim, Sun-Ju Yi, and co-workers at Chungbuk National University in South Korea have reviewed recent data on how cells regulate gene activity under hypoxic conditions. Advances in sequencing technology have allowed genome-wide studies of how hypoxia affects DNA structure and gene activation, revealing that gene-specific modifications may be more important than genome-wide modifications. Hypoxia is implicated in several diseases, such as cancer and chronic metabolic diseases, and a better understanding of how it affects gene activation may help identify new treatments for hypoxia-related diseases.

## Introduction

Appropriate oxygen concentrations are essential to the survival of living organisms. In eukaryotes, oxygen is essential for cellular respiration, which is closely related to energy metabolism; therefore, long-term exposure to low oxygen availability (hypoxia) is fatal. Hypoxic conditions occur during physiological and pathological processes, including embryogenesis, stem cell homeostasis, cancer, cardiovascular disease, lung disease, and anemia. Hypoxia is also caused by environmental stresses, such as vigorous exercise, high altitude exposure, or breath-hold diving^1,2^. Hypoxia induces adaptive cellular responses, such as changes in metabolism, including the transition from oxidative phosphorylation to glycolysis, induction of autophagy, inhibition of protein translation, and cell cycle progression; in addition, erythropoiesis and angiogenesis are triggered as physiological outcomes of hypoxia^2,3^. These responses are mediated primarily by the proteins of the hypoxia-inducible factor (HIF) family, which are master hypoxic regulators modulating the transcription of hundreds of hypoxia-related genes^4,5^.

HIFs act as heterodimers consisting of HIFα (HIF-1α, HIF-2α, and the less-characterized HIF-3 α) and HIFβ (also called aryl hydrocarbon receptor nuclear translocator, or ARNT). The levels and activities of HIFs are regulated by the hydroxylation of specific residues in these proteins; the hydroxylation reaction is catalyzed by HIF hydroxylases, members of the 2-oxoglutarate- and oxygen-dependent dioxygenase family that include prolyl hydroxylases (PHDs) 1–3 and factor inhibiting HIF (FIH)^6,7^. Under normoxic conditions, the proline residues in the oxygen-dependent degradation (ODD) domain in HIFα are hydroxylated by PHDs and bind to the Von Hippel Lindau (VHL) E3 ligase complex, thereby targeting HIFα for ubiquitination and proteasomal degradation or autophagy-mediated degradation. FIH represses HIFα transactivity by dissociating coactivator p300/CBP from HIFα via hydroxylation of an asparagine residue in the C-terminal transactivation domain of HIFα^8^. Hypoxia decreases the activity of oxygen-dependent HIF hydroxylases, thus maintaining the level and transactivity of HIFα^2,3^.

In eukaryotes, DNA is wrapped around histone octamers comprising two H2A-H2B dimers and an H3-H4 tetramer to form nucleosomes that make up chromatin^9^. The nucleosome compaction degree, density, and distribution determine gene expression. Regarding transcription, there are two main chromatin states: heterochromatin (repression) and euchromatin (activation). Histone cleavage, modification, remodeling, and eviction modulate the chromatin state^10–13^. Histone modification, one of the most extensively studied processes, includes methylation, acetylation, phosphorylation, ubiquitination, propionylation, and crotonylation. Histone modifications are regulated by specific histone-modifying enzymes, the so-called writers and erasers, and mostly occur at the N-terminal tail. Transcriptional states, such as activation and repression, are determined by the type and location of histone modifications. In addition, nonhistone proteins, including HIFα, p53, p65, and tubulin, can be modified by histone-modifying enzymes. Numerous studies have shown that hypoxia influences the activity of histone-modifying enzymes, which modulate the posttranslational modification of histones as well as nonhistone proteins^1,14^. This review discusses the recent findings regarding the roles of histone-modifying enzymes in histone and nonhistone protein modifications during hypoxia.

## Histone methylation in hypoxia

### Histone methyltransferases and demethylases

Histone methylation generally occurs at lysine and arginine residues. The well-known lysine methylation sites include H3K4, H3K9, H3K27, H3K36, H3K79, and H4K20, and arginine methylation sites include H3R2, H3R8, H3R17, H3R26, and H4R3^15^. The transcriptional effects of histone methylation depend on the methylation site. For example, methylation of H3K4, H3K36, H3K79, and H3R17 is found in transcriptionally active regions, whereas methylation of H3K9, H3K27, and H4K20 is found in transcriptionally repressed regions.

The various histone methyltransferases responsible for these types of methylation are categorized into three families: the SET (Su[var]3–9, Enhancer of Zeste, Trithorax) domain family, the DOT1L (DOT1-like) family, and the PRMT (protein arginine N-methyltransferase) family^15,16^. The SET domain family is divided into four subfamilies, namely, SUV39, SET1, SET2, and RIZ, as well as others that remain unclassified. Specifically, the SUV39 subfamily includes SUV39H1/2 and ESET for H3K9me2/3 and G9a/GLP for H3K9me1/2. In addition, the SET1 family includes SETD1A, SETD1B, and MLL1–4 for H3K4me1/2/3 and EZH1/2 for H3K27me2/3. In addition, the representative members of the SET2 family are NSD1 and NSD2, which are responsible for H3K36me3 and H3K36me2/3, respectively. Moreover, the nonclassified members, such as the SMYD subfamily, SUV4–20 subfamily, and SET7/9, are related to H3K4me2/3, H4K20me2/3, and H3K4me1, respectively. Furthermore, DOT1L is the only member of the DOT1L family catalyzing H3K79 methylation^17^. Last, the PRMT family is divided into 3 types: types I, II, and III. The type I PRMTs are PRMT1, 2, 3, 4, 6, and 8, which facilitate monomethylation and asymmetric dimethylation, whereas the type II PRMTs are PRMT5 and PRMT9, which perform mono- and symmetric dimethylation. PRMT7, the only member of the type III PRMT subgroup, is responsible for arginine monomethylation^17^.

Histone demethylases or lysine demethylases (KDMs), which contribute to protein methylation homeostasis, can be classified into 2 families—lysine-specific demethylases (LSDs) and JmjC domain-containing histone demethylases (JMJC demethylases)—according to their catalytic mechanisms^16,18^. The LSD family, containing LSD1 and LSD2, exhibits flavin adenine dinucleotide-dependent amine oxidation activity for catalytic reactions. LSD1 demethylates H3K4me1/2 and H3K9me1/2 in concert with interacting proteins, whereas LSD2 demethylates only H3K4me1/2. In contrast, JMJC demethylases are members of the 2-oxoglutarate-dependent dioxygenase superfamily; they use Fe^2+^ and oxygen to remove methyl groups through hydroxylation. KDM2–6 belong to the JMJC demethylase family. The KDM2 subfamily members, KDM2A and KDM2B, demethylate H3K36me2, while KDM2B demethylates H3K4me3. In addition, the KDM3 subfamily members, KDM3A and KDM3B, remove H3K9me1/2. Moreover, the KDM4 subfamily members, KDM4A-D, are involved in removing H3K9me2/3 and H3K36me2/3. Furthermore, the KDM5 subfamily members, KDM5A-D, are responsible for the demethylation of H3K4me2/3. Last, the KDM6 family members, KDM6A and KDM6B, are related to the removal of H3K27me2/3^18^.

### Effects of histone methyltransferases under hypoxic conditions

The SET domain family plays a major role in regulating gene expression in response to hypoxia (Tables 1 and 2)^19^. Among the SET domain family members, G9a/GLP has been extensively studied regarding its function under hypoxic conditions^20^. However, the role of G9a/GLP in transcription under hypoxic conditions remains controversial. G9a/GLP activates or represses hypoxia-inducible genes depending on the target of methylation. For example, Reptin and Pontin are chromatin remodeling factors methylated by G9a during hypoxia. Reptin methylated at Lys67 binds to the promoters of hypoxia-responsive genes, such as *PGK1* and *VEGF*, and represses the transcription of these genes, resulting in negative regulation of hypoxic responses^21^. On the other hand, methylation of Pontin by G9a/GLP under hypoxic conditions increases the recruitment of p300 and HIF-1α to the promoters of HIF-1α target genes, including *Est1*, thereby activating the expression of these target genes^22^. According to Bao et al., HIF-1α is methylated by G9a under hypoxic conditions; methylation at Lys674 of HIF-1α inhibits its transactivation domain activity, repressing the transcription of *NDNF* and *SLC6A3*^23^. Another study revealed that hypoxia increases G9a stability by reducing prolyl hydroxylation-mediated G9a degradation. Then, G9a suppresses transcription under hypoxic conditions by promoting H3K9 methylation in the promoter region of tumor suppressor genes, including *HHEX*, *GATA2*, and *ARNTL*^24^.

**Table 1:** Histone methylation in hypoxia.

Name	Substrate	Enzyme expression/activity in hypoxia	Target gene	Effect	Cell type (cell line)	O_2_ (%)	Ref.
*Histone methyltransferases*							
MLL1	H3K4		*HIF-2α*	Activation of *HIF-2α* expression	Glioma stem cell (387 GSC, 4302 GSC)	1	^25^
SETD1B	H3K4		*CA9, PHD3, VEGF*	Activation of hypoxia-inducible genes	Cervical (HeLa), lung (A549), and renal (786-O) cancer	1	^26^
G9a/GLP	H3K9		*HHEX, GATA2, ARNTL*	Repression of tumor suppressor genes	Breast cancer (MCF7)	1	^24^
EZH2	H3K27		*E-cadherin, p16INK4A*	Repression of *E-cadherin* and *p16* expression	Pancreatic cancer (SW1990)	1	^27^
*Histone demethylases*							
LSD1	H3K9me1/me2		*MTA1*	Activation of *MTA1* expression	Breast cancer (MDA-MB-231)	1 or CoCl_2_	^34^
KDM3A	H3K9me1/me2	Overexpressed/maintained	*PSA* enhancer	Activation of *PSA* expression	Prostate cancer (LNCaP)	<0.5, 3	^40^
KDM3A	H3K9me2	Overexpressed/maintained	*SLC2A3(GLUT3)*	Activation of *SLC2A3* expression	Endothelial cell (HUVECs)	1	^41^
KDM4A	H3K9me3	Maintained	*HIF-1α*	Activation of *HIF-1α* expression under mild hypoxic conditions	Colon carcinoma (RKO)	2	^42^
		Inactivated		Suppression of *HIF-1α* expression under severe hypoxic conditions		<0.1	
KDM4B	H3K9me3	Overexpressed/maintained	*SLC2A1, ELF3, IFI6, UCA1*	Activation of hypoxia-inducible genes	Colorectal cancer (SW480, HCT116)	1	^44^
KDM4C	H3K9me3	Overexpressed/maintained	*BNIP3*, *LDHA*, *PDK1*, *SLC2A1, LOXL2*, *L1CAM*	Activation of HIF-1α target genes	Breast cancer (MDA-MB-435), cervical cancer (HeLa)	1	^45^
KDM5A	H3K4me3	Inactivated	*BNIP3L*, *KLF10*	Activation of hypoxia-inducible genes	Cervical cancer (HeLa), skin fibroblast (HFF)	1	^47^
KDM5A	H3K4me3	Not changed/inactivated	*HMOX1*, *DAF*	Activation of *HMOX1*, *DAF* genes	Bronchial epithelial (Beas-2B), lung carcinoma (A549)	1	^48^
KDM6A	H3K27me3	Inactivated	*Actc1*, *Myl1*, *Myog*	Suppression of myogenic differentiation genes	Myoblast (C2C12)	2	^49^
KDM6A	H3K27me3	Overexpressed/maintained	*Ncx*	Activation of *Ncx* expression	Primary cardiomyocyte	5	^50^
						1	
KDM6B	H3K27me3	Overexpressed/maintained	*VEGFA*	Activation of *VEGFA*	Endothelial cell (HUVEC)	1	^51^

**Table 2:** Nonhistone methylation in hypoxia.

Name	Substrate	Target gene	Effect	Cell type (cell line)	O_2_ (%)	Ref.
*Histone methyltransferases*						
G9a	Reptin K67	*PGK1, VEGF*	Repression of HIF-1α target genes	Breast cancer (MCF7)	Not specified	^21^
	Pontin	*Est1*	Activation of HIF-1α target genes	Breast cancer (MCF7)	1	^22^
	HIF-1α K674	*NDNF, SLC6A3*	Repression of HIF-1α transactivity	Glioblastoma (U251MG)	1	^23^
SETD7	HIF-1α K32 HIF-2α K29	*LDHA, PDK, VEGF*	Reduction in SETD7 expression leading to activation of HIF-1α transactivity and HIF-1α stability	Renal carcinoma (RCC4), fibroblast (MEF)	1	^29^
SETD3	FOXM1	*VEGF*	Reduction in SETD3 expression leading to activation of *VEGF* expression	Cervical cancer (HeLa), glioblastoma (U-87MG)	1 or CoCl_2_	^32^
*Histone demethylases*						
LSD1	HIF-1α K32me1	Not determined	Increase in HIF-1α stability	Mouse embryonic fibroblast (MEF), cervical cancer (HeLa)	1	^31^
	HIF-1α K391me1			Breast cancer (MDA-MB-231)	1 or CoCl_2_	^34^

Several HMTs in the SET1 family are also associated with hypoxic responses. According to Heddleston et al., hypoxia-induced MLL1 increases the expression of HIF-2α, and inhibition of MLL1 decreases H3K4m3 levels while increasing H3K27m3 levels. These findings indicate that MLL1 regulates HIF-2α transcription via histone modification^25^. A recent study showed that SETD1B contributes to the activation of hypoxia-inducible genes. SETD1B associated with the HIF complex can be localized in the promoter region of hypoxia-related genes, such as *CA9*, *PHD3*, and *VEGF*, increasing the H3K4me3 levels at these loci. Therefore, in response to hypoxia, the HIF complex recruits the H3K4 methyltransferase SETD1B to facilitate the transcription of HIF target genes^26^.

In addition, EZH2 is involved in TWIST-induced epithelial-mesenchymal transition (EMT) under hypoxic conditions in pancreatic cancer cells. Hypoxia increases TWIST expression, which represses the transcription of *E-cadherin* and *p16INK4A*. TWIST overexpressed due to hypoxia interacts with EZH2 and Ring1B and binds to the promoters of *E-cadherin* and *p16INK4A*, increasing H3K27me3 and H2AK119ub1 in the promoter of *E-cadherin*^27^. In addition, under normoxic conditions, EZH2 modulates the expression of HIF-1α through H3K27 methylation in the promoter of HIF-1α. Furthermore, EZH2 is guided to the HIF-1α gene promoter via the lncRNA *HITT*, a hypoxia-responsive lncRNA whose expression decreases with increasing hypoxia. Thus, as *HITT* is downregulated under hypoxic conditions, the recruitment of EZH2 to the promoter of HIF-1α is reduced, increasing the expression of HIF-1α^28^.

SETD3 and SETD7, other members of the SET domain-containing methyltransferase family, are also known for their regulation of gene expression under hypoxic conditions^29,30^. Under normoxic conditions, SETD7 methylates HIF-1α at K32, blocking the transcriptional activity of HIF-1α and in turn repressing the expression of HIF-1α target genes, including *LDHA*, *PDK*, and *VEGF*^29^. Furthermore, methylation of HIF-1α at K32 by SET7/9 in the nucleus decreases the stability of HIF-1α^31^. Under hypoxic conditions, the SET7 protein level is reduced, which increases the stability and transactivity of HIF-1α, thereby inducing the expression of HIF-1α target genes. In addition, SETD3 is a negative regulator of VEGF expression during hypoxia^32,33^. Moreover, SETD3 interacts with and methylates FOXM1, which binds to the promoter of *VEGF*. Hypoxia decreases the SETD3 level, leading to the disassociation of SETD3 and FoxM1 from the VEGF promoter.

Taken together, these observations indicate that HMTs differentially regulate gene expression in hypoxia by methylating nonhistone proteins and histones. It is likely that methylation of nonhistone proteins typically affects their stability and/or interaction with other proteins (e.g., transcription factors). Moreover, hypoxia drives HMTs to cooperate with transcription factors or lncRNAs to control histone methylation. Eventually, these methylation events may modulate gene expression, leading to adaptation to cellular hypoxia.

### Effects of histone demethylases under hypoxic conditions

According to a growing body of evidence, hypoxia affects the gene expression and functions of histone demethylases (Tables 1 and 2)^2^. For example, LSD1 affects hypoxic responses by demethylating HIF-1α and histones. The demethylase activity of LSD1 toward HIF-1α facilitates HIF-1α stabilization by inhibiting VHL-induced HIF-1α degradation. Recent studies have shown that LSD1 demethylates HIF-1α at K32 and K391 in response to hypoxia-mimicking conditions^31,34^. In addition, pharmacological inhibition or siRNA-mediated silencing of LSD1 expression effectively reduces the HIF-1α protein level^35^. Furthermore, LSD1 increases the transcription of *MTA1* via H3K9 demethylation in the promoter region of *MTA1*, enhancing NuRD complex-mediated deacetylation of HIF-1^34^.

JMJC demethylases require oxygen to remove methyl groups. The results of many studies indicate that some JMJC demethylases are inactivated as oxygen availability decreases and that their expression is upregulated to compensate for the reduced enzymatic activity^1,36^. KDM3A and KDM4B are upregulated via HIF-1α in hypoxia^36–38^. Although the expression of KDM3A and KDM4B is induced by hypoxia, the levels of H3K9me2 and H3K9me3 are unchanged or even increased^37,39^. Chromatin immunoprecipitation assays in macrophages revealed increases in repressive marks H3K9me2 and H3K9me3 in the specific promoter regions of *Ccl2*, *Ccr1*, and *Ccr5* that resulted in decreases in their expression under hypoxic conditions (1% O_2_)^39^. These results suggest that hypoxia suppresses the demethylase activity of KDM3A and KDM4B while increasing their expression levels. In contrast, another study revealed that hypoxia in prostate cancer cells increased the expression of KDM3A and that its catalytic activity was maintained under severe hypoxic conditions (0.5% O_2_)^40^. KDM3A occupies the *PSA* enhancer region, demethylating H3K9me1 and H3K9me2. This recruits p300 and MLL4, thereby resulting in the addition of active histone marks (i.e., H3K9ac and H3K4me3) and increased gene expression. Furthermore, Mimura et al. reported that HIF-1 and KDM3A upregulate glycolytic genes in response to hypoxia (1% O_2_) independent of cell type^41^. In particular, KDM3A is recruited to the *SLCA3* locus in a HIF-1-dependent manner and demethylates H3K9me2. In some cases, the KDM4 subfamily members, which also regulate HIF genes, exhibit increased expression levels and are functional in hypoxia. According to Dobrynin et al., KDM4A stimulates the expression of HIF-1α by removing a methyl group from H3K9me3 at the *HIF-1α* locus under mild hypoxic conditions (2% O_2_). Loss of KDM4A decreases the *HIF-1*α mRNA level and HIF-1α protein stability, thus reducing the HIF-1α level^42^. However, KDM4A demethylase activity is abolished under more severe hypoxic conditions (less than 0.1% O_2_). Similarly, Hancock et al. showed that KDM4A enzymatic activity decreased gradually with oxygen depletion (0.1–5% O_2_)^43^. These results suggest that KDM4A acts as an oxygen sensor. KDM4B expression is induced in a HIF-1α-dependent manner under hypoxic conditions (1% O_2_); it upregulates the expression of a subset of hypoxia-inducible genes by decreasing H3K9me3 in their promoters^44^. KDM4C expression is also induced under hypoxic conditions (1% O_2_). KDM4C selectively interacts with HIF-1α, which mediates the recruitment of KDM4C to the HREs in HIF-1 target genes, allowing KDM4C to decrease H3K9me3 and promote the binding of HIF-1 to the HREs, thereby activating the transcription of *BNIP3*, *LDHA*, *PDK1*, and *SLC2A1*^45^. These results suggest that KDM4 demethylase activity is maintained or decreased based on the hypoxia status.

Some histone demethylases, such as KDM5A, KDM6A, and KDM6B, act as direct oxygen sensors^46^. According to Batie et al., KDM5A inactivation under hypoxic conditions (1% O_2_) is related to hypermethylation of H3K4 in cancer cells. In addition, hypoxia causes a rapid increase in global histone methylation independent of HIF. KDM5A, upon sensing low oxygen levels under hypoxic conditions, becomes enzymatically inactivated, thus inhibiting the removal of a methyl group from H3K4me3 in the promoters of hypoxia-inducible genes, such as *BNIP3L* and *KLF10*^47^. Consistent with this finding, KDM5A demethylase activity is decreased during hypoxia (1% O_2_) in lung cancer cells, which increases the H3K4me3 levels in the promoters of the *HMOX1* and *DAF* genes^48^. Chakraborty et al. also showed that KDM6A senses oxygen, determining cell fate. They found that hypoxia (2–5% O_2_) induces HIF-independent hypermethylation at H3K27. Hypoxia blocks C2C12 cell myogenic differentiation, which is not due to HIF activation and 2-hydroxyglutarate. Similar to their effects on hypoxia, treatment with the KDM6 family inhibitor GSK-J4 and knockdown of *KDM6A* inhibited myogenic differentiation and increased the level of H3K27me3, a repressive mark. During muscle differentiation, the reduction in H3K27me3 at late myogenic genes, such as *Actc*, *Myl1*, and *Myog*, is blunted by hypoxia. These results suggest that KDM6A inactivation by hypoxia increases H3K27me3 levels and inhibits transcriptional activation of genes involved in differentiation^49^. On the other hand, Li et al. reported that hypoxia (1% O_2_) in cardiomyocytes significantly upregulates KDM6A expression, which increases the expression of *Ncx*, encoding the Na+/Ca_2_+ exchanger, by reducing the H3K27me3 level in the *Ncx* promoter, thus decreasing intracellular calcium influx^50^. Liu et al. showed that hypoxia (1% O_2_) induces KDM6B expression, which elevates *VEGF* gene expression and angiogenesis via removal of H3K27me3^51^. However, another study reported that severe hypoxia (0.1% O_2_) increases genome-wide H3K27me3, suggesting that the changes in the chromatin state in response to hypoxia are due to the inactivation of KDM6B^52^.

Considering these findings, it is clear that hypoxia increases the expression of some JMJC demethylases. However, the demethylase activity of JMJC demethylases such as KDM3–6 under hypoxic conditions is still incompletely understood. Further studies will be needed to clarify this issue.

## Histone acetylation in hypoxia

### Histone acetyltransferases and deacetylases

Histone acetyltransferases (HATs) induce acetylation of the amino group of lysine residues in histone tails and even in nonhistone proteins. Histone acetylation diminishes the interaction between histones and DNA due to the neutralization of the lysine residue, opening the chromatin structure and subsequently inducing transcriptional activation^15,53^. Nuclear-localized HATs can be classified into five families based on their conserved catalytic domains and structural similarities: GNAT (Gcn5-related N-acetyltransferases), p300/CBP, HAT1, Rtt109, and MYST (MOZ, yeast YBF2, SAS2, and TIP60)^15^. These HATs are responsible for histone acetylation at H3K9, H3K14, H3K27, H4K8, and H4K12. The GNAT family comprises GCN5 (general control of amino acid synthesis 5), PCAF (p300/CBP associated factor), and ELP3 (elongation protein 3), each of which contains a bromodomain and a coactivator binding domain. The p300/CBP family comprises p300 and CBP, each of which contains a bromodomain and a PHD finger. The MYST family comprises TIP60 (TAT interacting protein 60), MOZ (monocytic leukemia zinc-finger protein), MOF (males absent on the first), MORF (monocytic leukemia zinc-finger protein-related factor), and HBO1 (human acetylase binding to ORC1); each of these members contains a chromodomain for protein–protein interaction^53^.

In contrast, histone deacetylases (HDACs) antagonize HATs by removing acetyl groups from various proteins via their conserved deacetylation domains. Based on domain structures and cofactors, HDACs can be divided into four classes: I, II, III, and IV. The class I, II, and IV HDACs use zinc ions as the cofactor for their catalytic activity, whereas the class III HDAC, the sirtuin protein, uses nicotinamide adenine dinucleotide. The class I HDACs, HDAC1, HDAC2, HDAC3, and HDAC8, are mainly localized in the nucleus and deacetylate histones. HDAC1 and HDAC2 act by forming large protein complexes, such as the CoREST (repressor element-1 silencing transcription corepressor), Sin3A (SWI-independent-3A), MiDAC (mitotic deacetylase), and NuRD (Mi2/nucleosome remodeling deacetylase) complexes. HDAC3 interacts with the NCoR/SMRT (nuclear receptor corepressor/silencing mediator for retinoid or thyroid-hormone receptors) complex. Unlike other HDACs, HDAC8 does not require additional proteins to catalyze deacetylation^54,55^. Class II HDACs have a conserved catalytic domain in the C-terminal region. They are subdivided into class IIa (HDAC4, HDAC5, HDAC7, and HDAC9) and class IIb (HDAC6 and HDAC10) based on the presence or absence of additional domains. Class IIa HDACs, for instance, contain an N-terminal domain that has a conserved serine residue for their nuclear export and other protein-binding motifs. Class IIb HDACs contain an extended C-terminal domain. A zinc-finger ubiquitin-binding domain and a leucine-rich repeat domain are found in HDAC6 and HDAC10, respectively. The class III HDACs, which are homologous to Sir2, constitute SIRT1 to SIRT7. Each of these SIRTs can be localized in the nucleus, cytoplasm, or mitochondria; they have various enzymatic activities, such as ADP ribosyltransferase (SIRT1), desuccinylase, and demalonylase (SIRT5) activities. Last, the only member of the Class IV HDAC family is HDAC11, which is homologous to Hos3. Unlike the other HDACs, HDAC11 has not been extensively characterized^54,55^.

### Effects of histone acetyltransferases under hypoxic conditions

Several studies have evaluated the roles of HATs in HIF-1α stability and hypoxia-induced transcriptional activity (Tables 3 and 4). The best-characterized acetyltransferases for HIF are p300 and CBP^56–58^. p300/CBP interacts with HIF-1α to recruit it to the promoter region of *EPO* and *VEGF*, inducing gene activation under hypoxic conditions^56^. Several studies have reported that p300/CBP acetylates HIF-1α at K709 and suppresses the polyubiquitination and degradation of HIF-1α under hypoxic conditions; as a result, p300/CBP increases HIF-1α transcriptional activity and cancer cell proliferation^59,60^. Among the GNAT family members, PCAF and ELP3 are implicated in hypoxic responses. During hypoxia, PCAF acetylates HIF-1α at K674, thus activating HIF-1α via p300 recruitment^61^. According to a recent study, ELP3 acetylates PAK1 at K420 in hypoxic environments, increasing the catalytic activity of PAK1 by inhibiting its dimerization and resulting in hypoxia-induced autophagy^62^.

**Table 3:** Histone acetylation in hypoxia.

Name	Substrate	Target gene	Effect	Cell type (cell line)	O_2_ (%)	Ref.
*Histone acetyltransferases*						
p300/CBP		*EPO, VEGF*	Activation of *EPO* and *VEGF* expression	Hepatocellular carcinoma (Hep3B)	1	^56^
TIP60	H3K9	*ANKRD37*	Activation of hypoxia-inducible genes	Colorectal cancer (HCT116)	1	^63^
*Histone deacetylases*						
HDAC1	H3ac	*lncRNA CF129*	Suppression of *lncRNA CF129*	Pancreatic cancer (PANC-1, BxPC-3)	1	^69^
		*lncRNA FAM99A*	Suppression of *lncRNA FAM99A*	Hepatocellular carcinoma (Hep3B, SK-Hep-1)	1	^70^
HDAC3	H3K4ac	*E-cadherin*	Suppression of epithelial genes	Hypopharyngeal carcinoma (FaDu), breast cancer (MCF7)	1	^73^
	H3K9ac	*miR-627–5p*	Suppression of *miR-627–5p*	Hepatocellular carcinoma (Hep3B, SMMC-7721)	1	^75^
HDAC9	H3K9ac	*Atg7, Beclin1, LC3*	Suppression of autophagy genes	Myoblast (C2C12)	1	^82^

**Table 4:** Nonhistone acetylation in hypoxia.

Name	Substrate	Effect	Cell type (cell line)	O_2_ (%)	Ref.
*Histone acetyltransferases*					
P300/CBP	HIF-1α K709	Increase in HIF-1α stability	Embryonic kidney cell (HEK293T)	1	^59^
			Glioblastoma (LN229)		^60^
PCAF	HIF-1α K674	Increase in HIF-1α stability	Fibrosarcoma (HT1080)	1	^61^
ELP3	PAK1 K420	Inhibition of PAK1 dimerization leading to enhanced PAK1 activity	Glioblastoma (LN229)	Not specified	^62^
MYST1	N-terminal of HIF-1α	Decrease in HIF-1α stability	Hepatocellular carcinoma (Huh-7, Hep3B)	CoCl_2_	^64^
*Histone deacetylases*					
HDAC2	HIF-1α K532	Increase in HIF-1α stability	Oral squamous cell carcinoma (HSC-3)	Not specified	^72^
HDAC3	P-TEFb	Inhibition of transcription elongation	Cervical cancer (HeLa)	0.5	^74^
HDAC4	K10, K11, K12, K19, and K21 of HIF-1α	Increase in HIF-1α stability	Hepatocellular carcinoma (Hep3Bc1), Prostate cancer (C42B)	1	^77^
HDAC5	HSP70	Increase in mature HIF-1α accumulation	Hepatocellular carcinoma (Hep3B), cervical cancer (HeLa)	1	^79^
HDAC6	HSP70	Increase in HIF-1α stability and transactivity in a VHL-independent manner	Embryonic fibroblast (MEF)	1	^81^
			Lung cancer (A549)	1	^80^
			Renal cell carcinoma (UMRC2)	(CoCl_2_)	^124^
SIRT1	HIF-1α K674	Suppression of HIF-1α target gene	Fibrosarcoma (HT1080)	1	^61^
			Renal proximal tubule cell (HK2)		^84^
SIRT1	HIF-2α K385, K685, and K741	Activation of HIF-2α transactivity	Hepatocellular carcinoma (Hep3B)	1	^57,85^
SIRT2	HIF-1α K709	Decrease in HIF-1α stability (Induction of HIF-1α degradation)	Cervical cancer (HeLa)	1	^86^
			B cell precursor leukemia (NALM-6)		^87^
SIRT3	FOXO3	Inhibition of FOXO3 degradation	Endothelial cell (HUVEC)	2	^88^

TIP60, a member of the MYST family, functions as a coactivator of HIF-1α, similar to PCAF. TIP60 interacts with HIF-1α and is recruited to HIF target genes, such as *ANKRD37*, under hypoxic conditions. Localized TIP60 not only acetylates H3K9 but also activates RNAPII^63^. Unlike other HATs, MYST1 contributes to hypoxia tolerance by downregulating HIF-1α. For example, MYST1 catalyzes the N-terminal acetylation of HIF-1α, inducing HIF-1α degradation under hypoxia-mimicking conditions. Thus, inactivation of MYST1 during hypoxia increases the expression and stability of HIF-1α^64^. These results imply that the differential effects of HIF-1α acetylation on its stability and transcriptional activity may originate from the acetylated residues or acetylases of HIF-1α.

### Effects of histone deacetylases under hypoxic conditions

Early studies have reported that several HDAC inhibitors, such as trichostatin A, sodium butyrate, valproic acid, and apicidin, decrease the stability and hypoxia-induced transcriptional activity of HIF-1α^65–67^. These results suggest that HDACs are involved in hypoxia (Tables 3 and 4). Among the class I HDACs, HDAC1 and HDAC3 interact with the ODD domain of HIF-1α, increasing the HIF-1α level under hypoxic conditions. Furthermore, HDAC1 represses the expression of p53, VHL, and RECK, increasing HIF-1α stability^65,68^. According to recent studies, the HDAC1–HIF-1α complex binds to lncRNA promoter regions, resulting in H3 deacetylation and decreased lncRNA expression under hypoxic conditions. Specifically, HDAC1 and HIF-1α are recruited to the promoter region of the *lncRNA CF129*, inhibiting the transcription of *CF129*, which induces the ubiquitination and degradation of p53. Thus, HDAC1-mediated downregulation of *CF129* causes the accumulation of p53, increasing the transcription of *FOXC2* and *HIF-1α*^69^. Hypoxia also reduces the expression of the *lncRNA FAM99A*, which inhibits EMT in hepatocellular carcinoma cells by negatively regulating *miR-92a*. Hypoxia-induced *FAM99A* downregulation is dependent on HIF-1α and HDAC1 via enhanced H3 deacetylation in the promoter of *lncRNA-FAM99A*. Thus, HDAC1 plays a central role in hypoxia-induced metastasis via the HIF-1α/HDAC1/FAM99A/miR-92a/E-cadherin axis^70^.

Similar to HDAC1, HDAC2 and HDAC3 are activated in hypoxia. HDAC2 is phosphorylated by CK2 under hypoxic conditions, leading to HDAC activation. Hypoxia-induced HDAC activation causes VHL downregulation and HIF-1α stabilization^71^. Moreover, HDAC2 deacetylates K532 in HIF-1α, whose acetylation is required for binding to VHL. Thus, deacetylation of HIF-1α at K532 increases the stability of HIF-1α, leading to migration and invasion of cancer cells^72^. Wu et al. demonstrated that HIF-1α-induced HDAC3 is required for hypoxia-induced EMT and metastasis in head and neck squamous cell carcinoma. During hypoxia, HDAC3 interacts with WDR5 and recruits the HMT complex to the promoter regions of mesenchymal genes, such as *N-cadherin* and *Vimentin*, inducing their expression via an increase in H3K4me2/me3. Concurrently, HDAC3 functions as a corepressor to suppress epithelial genes, such as *E-cadherin*, through deacetylation of H3K4ac^73^. In addition, HDAC3 is involved in repressing transcription elongation during hypoxia. HDAC3 deacetylates p-TEFb and promotes the formation of an inactive complex of p-TEFb and its inhibitor HEXIM1, inhibiting transcription elongation under acute hypoxic conditions^74^. A recent study suggested that hypoxia-induced HDAC3 increases hepatocellular carcinoma progression by inhibiting the expression of the microRNA *miR-627–5p*, which targets Bcl3. The hypoxia-induced decrease in the expression of *miR-627–5p* is dependent on HDAC3-mediated H3K9ac acetylation of its promoter region^75^. Some class II HDACs, including IIa and class IIb HDACs, are also associated with HIF-1α stability in a VHL-independent manner during hypoxia. HDAC4 interacts with HIF-1α and promotes its stability in VHL-deficient cells^76^. HDAC4 deacetylates multiple lysine residues, such as K10, K11, K12, K19, and K21, in HIF-1α to increase its stability, suggesting a potential role of HDAC4 in hypoxia-induced glycolysis^77^. Another study reported that HDAC4 and HDAC5 associate with the inhibitory domain of HIF-1α and increase its transactivation function by inducing p300 binding^78^. In addition, HDAC5 deacetylates HSP70, subsequently enhancing the formation of a complex of HIF-1α and HSP90 and causing rapid HIF-1α accumulation in the nucleus. Moreover, under hypoxic or low-glucose conditions, AMPK-mediated cytosolic translocation of HDAC5 increases HIF-1α maturation through deacetylation of HSP70 and the interaction between the HSP90 complex and HIF-1α, activating transcriptional function of HIF-1α in the nucleus^79^. HDAC6 also enhances the stability and transcriptional activation of HIF-1α in a manner similar to that of other HDACs^76,80,81^. Moreover, HDAC9 inhibits myoblast differentiation under hypoxic conditions. Hypoxia induces a significant increase in HDAC9 expression in myoblasts. HDAC9 is recruited to the promoter regions of autophagy-related genes, including *Atg7*, *Beclin1*, and *LC3*, and reduces their H3K9ac levels, disrupting the regulation of autophagy in myoblasts^82^.

Class III HDACs exert various effects under hypoxic conditions. SIRT1 deacetylates K674 in HIF-1α and K741 in HIF-2α^61,83^. On the one hand, deacetylation of HIF-1α by SIRT1 disrupts its interaction with p300 and represses HIF-1 target genes, suggesting that SIRT1 is a negative regulator of HIF-1α^61,84^. On the other hand, SIRT1 also interacts with HIF-2α and deacetylates HIF-2α at K741. However, SIRT1 enhances the transcriptional activity of HIF-2α during hypoxia^57,85^. SIRT2 induces deacetylation of K709 in HIF-1α and increases the binding affinity of HIF-1α for prolyl hydroxylase 2, leading to HIF-1α hydroxylation and degradation^86,87^. In addition, SIRT3 is associated with cell survival and angiogenesis in endothelial cells under hypoxic conditions. The increased SIRT3 resulting from hypoxia deacetylates FOXO3, reducing its phosphorylation, ubiquitination, and degradation. As a result, the inhibitory effect of SIRT3 on FOXO3 degradation increases the levels of mitochondrial antioxidant enzymes and reduces the accumulation of mitochondrial ROS during hypoxia^88^. Moreover, SIRT6 enhances the expression and stability of HIF-1α, increasing EMT in papillary thyroid cancer^89^. SIRT6 also increases HIF-1α expression by inhibiting the ubiquitin-mediated proteasomal degradation of HIF-1α, thus regulating angiogenesis in HUVECs^90^. Interestingly, SIRT7 interacts with HIF-1 and HIF-2 and inhibits the transcriptional activity of HIF genes in a noncatalytic manner^91^. Although many studies have found that SIRTs are involved in cellular responses under hypoxic conditions, the detailed mechanisms by which they affect hypoxic responses should be investigated.

## Genome-wide landscapes of histone modifications and chromatin structures under hypoxic conditions

Advances in next-generation sequencing technology have allowed researchers to examine genome-wide profiles of chromatin structures and gene regulation. Many studies have sought to identify the effects of hypoxia on histone modifications and genome-wide chromatin dynamics using chromatin immunoprecipitation sequencing (ChIP-seq) and assay for transposase-accessible chromatin with sequencing (ATAC-seq).

As hypoxia has been shown to induce global histone methylation, most studies have focused on changes in histone marks such as H3K4me3, H3K9me3, and H3K27me3. Hypoxia-induced changes in H3K4me3 and H3K27me3 marks are transient, and these marks return to basal levels upon reoxygenation in MCF7 cells^52^. H3K27me3 changes more dynamically under hypoxic conditions than H3K4me3. Interestingly, hypoxia-induced bivalency, the installation of H3K27me3 at H3K4me3-marked loci, occurs predominantly near transcription start sites and overlaps with bivalent genes in embryonic stem cells. Similarly, Adriaens et al. demonstrated that hypoxia-induced bivalency, i.e., marking with both H3K4me3 and H3K27me3, is associated with CpG-rich regions near developmental genes in MCF7 cells^92^. These bivalent marks dampened transcriptional fluctuation upon repeated exposure to hypoxia, suggesting that transcriptional activity at these loci is tightly controlled by H3K27me3 demethylases and their cofactors. In addition, genomic profiling of H3K4me3, H3K9me3, and H3K27me3 was performed in adipocyte-derived stem cells under hypoxic conditions^93^. Differentially expressed genes (DEGs) and differentially methylated genes were observed during hypoxia. Interestingly, each group of DEGs was predominantly associated with alterations in a single type of histone trimethylation rather than with complex combinatorial changes in histone methylation. Furthermore, a diverse set of transcription factors, coupled with histone methylation, fine-tune gene expression under hypoxic conditions.

Several studies have examined the chromatin accessibility landscape in response to oxygen fluctuations (hypoxia and reoxygenation) using ATAC-seq^94–98^. For example, it has been shown that hypoxia promotes neuroblastoma cell dedifferentiation by decreasing the cellular acetyl-CoA level and, subsequently, global histone acetylation^96^. ATAC-seq analysis revealed that global hypoacetylation under hypoxic conditions significantly reduces chromatin accessibility at RAR/RXR binding sites, blocking the RA response and cell differentiation. Wang et al. investigated the temporal profiles of the transcriptome and chromatin accessibility occurring in HL-1 cardiomyocytes in response to hypoxia (for 4 and 8 h) and reoxygenation (for 24 h)^95^. They found a total of 2912 DEGs and 3004 differential peaks. While hypoxia-related genes, including VEGF, Angpt1, Slc2a1, Bnip3, and Casp3, showed a positive relationship between chromatin structure and gene expression, some ATAC-seq data showed negative or rare correlations with gene expression. On the other hand, recent ATAC-seq analyses support the idea that hypoxia induces locus-specific changes in chromatin accessibility. Hypoxic conditions in HeLa cells have been shown to locally alter chromatin structures via a mechanism that is predominantly HIF dependent, indicating that HIF plays an important role in altering chromatin accessibility during hypoxia^94^. H3K4me3 is specifically increased at HIF binding sites. Although further studies are needed to elucidate the underlying mechanisms by which HIF promotes euchromatinization under hypoxic conditions, these results suggest that HIF might recruit coactivators such as HMT (i.e., SETD1B) or chromatin remodeling factors.

## Pathophysiological implications of histone-modifying enzymes in hypoxia-associated disorders

Prolonged or intermittent hypoxia (IH) causes alterations in gene expression and cellular functions, leading to pathological consequences. A growing body of evidence has demonstrated that histone-modifying enzymes play important roles in the pathogenesis of several hypoxia-related disorders, such as cancers, brain injuries, myocardial ischemia, metabolic diseases, and chronic heart and kidney diseases^99,100^. Therefore, expanding our understanding of the epigenetic dysregulation associated with hypoxia may provide therapeutic opportunities for these diseases.

Hypoxia is a typical feature in the microenvironment of most solid tumors^101^. In response to hypoxia, cancer cells activate anaerobic glycolysis, EMT, angiogenesis, invasion, metastasis, and drug resistance, which are critical for cancer cell survival and cancer progression^36,102^. Many histone-modifying enzymes, as listed in Tables 1–4, play key roles in the altered expression of genes associated with angiogenesis, anaerobic glycolysis, and EMT under hypoxic conditions.

Renal hypoxia is associated with several pathologies, including tubular injury, microvascular injury, inflammation, and fibrosis, thus contributing to chronic kidney disease (CKD) progression^103,104^. Persistent mitochondrial fission is a characteristic of sustained tubular damage after renal ischemia–reperfusion injury^105^. HDAC8 protects human renal proximal tubular cells against mitochondrial dysfunction induced by cobalt and hypoxia/reoxygenation (H/R). HDAC8 suppresses the gene expression of *DRP1*, a key regulator of mitochondrial fission, by removing H3K27ac in the promoter region of *DRP1*^106^. These results suggest that failure of the protective role of HDAC8 in H/R-induced cytotoxicity may be related to CKD progression.

Tissue hypoxia is caused by poor circulation and insufficient oxygen delivery and is associated with several cardiovascular disorders, including atherosclerosis, pulmonary arterial hypertension, and heart failure^107–109^. Because alterations in histone modifications are frequently found in cardiovascular diseases^110^, the pathophysiological relevance of histone-modifying enzymes in hypoxia-induced cardiovascular diseases has been studied. It has been shown that induction of acute myocardial infarction (AMI) in rats and hypoxia in cardiomyocytes facilitates the expression and enzymatic activity of KDM6A^50^. Knockdown of *Kdm6a* dramatically induces cardiomyocyte apoptosis under hypoxic conditions, suggesting that KDM6A plays an important role in AMI development.

Hypoxic brain injury is caused by stroke, cardiac or respiratory arrest, low blood pressure, high altitude exposure, suffocation, asphyxia, poisoning, and drug overdose. The neurons in the brain require a continuous supply of oxygen to survive and function. If the oxygen supply is interrupted, brain function is immediately disrupted, and neurons begin to die within 5 min. In particular, perinatal hypoxia can occur during pregnancy, labor, and birth, contributing to neurological dysfunction. Perinatal hypoxic-ischemic brain damage is known to be associated with epigenetic dysregulation^111^. Recently, Xue et al. reported that sevoflurane exerts neuroprotective effects against hypoxia-ischemia through inhibition of activated autophagy in the hippocampus in neonatal rats^112^. They observed that hypoxia-ischemia decreases the levels of pAKT, mTOR, EZH2, and H3K27me3, which are restored by sevoflurane. The EZH2 inhibitor GSK126 significantly reverses sevoflurane-mediated long-term neurological protection in neonatal rats. Although the exact mechanisms by which EZH2-mediated H3K27me3 affects the transcriptional regulation of autophagy-related genes remain to be fully understood, these results suggest that EZH2-mediated H3K27me3 is involved in the regulation of autophagy-related genes in the response to hypoxia-ischemia and in the neuroprotection mediated by sevoflurane.

Several studies have observed upregulation of HDACs in perinatal hypoxic-ischemic injury. In rats, fetal asphyxia caused increased expression of class I and II HDACs, including HDAC1, HDAC2, HDAC3, HDAC10, and HDAC11^113,114^. Furthermore, HDAC inhibitors are neuroprotective and neurogenic in adult ischemia models and immature perinatal brains with hypoxic-ischemic injury^115–119^. These results suggest that treatment of perinatal hypoxia with HDAC inhibitors results in the accumulation of acetylated proteins, which modulate gene expression, leading to inhibition of neuronal cell death and promotion of neurogenesis.

## Conclusion

**Fig. 1:**
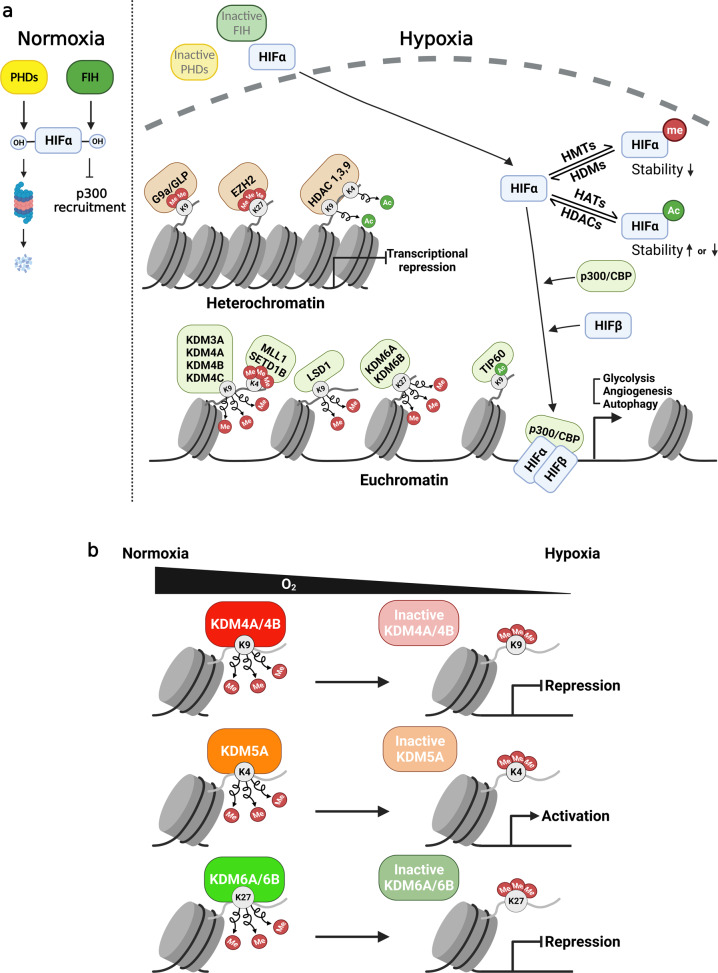
Mechanisms of hypoxic gene expression in the context of chromatin structure. **a** In normoxia, HIFα is subjected to oxygen-dependent prolyl hydroxylation via PHDs, leading to its proteasomal degradation. FIH inhibits HIFα signaling by hydroxylating an asparagine residue in HIFα and dissociating the HIFα–p300 complex. Under hypoxic stress, HIFα is stabilized mainly via the inactivation of PHDs and FIH. It then translocates to the nucleus to form a heterodimer with HIFβ, which binds to hypoxia response elements (HREs), increasing gene transcription. The stability and transactivity of HIFα are further modulated by its acetylation and methylation. Under hypoxic conditions, histone-modifying enzymes dynamically change the chromatin structure. Some HMTs (e.g., G9a and EZH2) and HDACs form heterochromatin by inducing repressive histone marks. In contrast, other HMTs (e.g., MLL1 and SETD1B), HDMs (e.g., LSD1, KDM3A, KDM4A-C, KDM6A, and KDM6B), and HATs (e.g., p300/CBP and TIP60) induce activating marks in chromatin, forming euchromatin. These events lead to the activation of hypoxia-related genes, including those associated with glycolysis, angiogenesis, and autophagy. **b** Some JMJC histone demethylases (e.g., KDM4A, KDM4B, KDM5A, KDM6A, and KDM6B) function as direct oxygen sensors. Enzymatic inactivation has been observed under specific hypoxic conditions and induces the formation of either heterochromatin or euchromatin.

Over time, many researchers have studied the adaptive mechanisms activated under hypoxic stress. Cells overcome hypoxic stress by expressing multiple hypoxia-related genes via chromatin remodeling. This review provides a comprehensive survey of the findings on the roles of histone-modifying enzymes in hypoxia (Fig. 1a). Hypoxia induces dynamic changes in the chromatin state by regulating histone-modifying enzymes. Hypoxia decreases the acetyl-CoA level, leading to a global reduction in histone acetylation. HDAC activation under hypoxic conditions further induces histone deacetylation to form heterochromatin. However, some HATs (e.g., p300/CBP and MYST) induce activating marks in the local chromatin region, forming euchromatin. Overall, hypoxia is likely to increase global histone methylation (e.g., H3K4, H3K9, H3K27, and H3K36) due to the induction of HMT activity and the reduction in histone demethylase activity (Fig. 1b). However, not all JMJC demethylases are inactivated under hypoxic conditions; some KDMs maintain their enzymatic activity under hypoxic conditions and activate HIF target genes by stabilizing HIF or removing repressive marks in the promoters of specific target genes. Moreover, in some cases, H3K4 HMTs (e.g., MLL1 and SETD1B), H3K9 HMTs (e.g., G9a), and H3K27 HMTs (e.g., EZH2) induce active or repressive marks in the local chromatin, where the corresponding HMTs are recruited by various transcription factors. These findings suggest that gene-specific changes in histone modifications may be more important than global histone modifications in modulating the expression of key genes associated with hypoxia. Therefore, a more detailed investigation of the interplay between histone modifications and gene expression is needed. Although genome-wide profiling has been described in this review, more studies using integrated next-generation sequencing technologies, such as ChIP-seq, ATAC-seq, Hi-C seq, and single-cell RNA-seq, will provide promising insights that will help us study this topic.

IH describes a condition of periodic alternating exposure to hypoxia and normoxia. IH occurs in many pathological conditions, such as obstructive sleep apnea or solid tumors; however, its biological effects are largely unknown. Interestingly, recent studies have shown that continuous hypoxia increases the levels of both HIF-1α and HIF-2α, whereas IH increases the HIF-1α level but decreases the HIF-2α level^120,121^. Unlike chronic hypoxia, IH activates lysine acetylation of H3 and HIF-1α by reducing HDAC3 and HDAC5 activity, enhancing HIF transactivity^122^. According to Martinez et al., IH enhances the expression of HIF-1α by increasing the expression level and enzymatic activity of KDM4A-C and demethylating H3K9me3 in the HIF-1α promoter^123^. Therefore, it will be interesting to examine the differential effects of these two oxygen-sensing pathways under chronic hypoxia and IH on histone modifications and gene expression.

Indeed, a better understanding of the epigenetic mechanisms related to normal and pathological oxygen conditions will help us develop innovative therapeutics for hypoxia-related diseases.

## References

[1] Kindrick JD, Mole DR (2020). Hypoxic regulation of gene transcription and chromatin: cause and effect. Int. J. Mol. Sci..

[2] Hancock RL, Dunne K, Walport LJ, Flashman E, Kawamura A (2015). Epigenetic regulation by histone demethylases in hypoxia. Epigenomics.

[3] Batie M, Del Peso L, Rocha S (2018). Hypoxia and chromatin: a focus on transcriptional repression mechanisms. Biomedicines.

[4] Majmundar AJ, Wong WJ, Simon MC (2010). Hypoxia-inducible factors and the response to hypoxic stress. Mol. Cell.

[5] Lee P, Chandel NS, Simon MC (2020). Cellular adaptation to hypoxia through hypoxia inducible factors and beyond. Nat. Rev. Mol. Cell Biol..

[6] Islam MS, Leissing TM, Chowdhury R, Hopkinson RJ, Schofield CJ (2018). 2-Oxoglutarate-dependent oxygenases. Annu. Rev. Biochem..

[7] Frost J, Frost M, Batie M, Jiang H, Rocha S (2021). Roles of HIF and 2-oxoglutarate-dependent dioxygenases in controlling gene expression in hypoxia. Cancers (Basel).

[8] Hewitson KS (2002). Hypoxia-inducible factor (HIF) asparagine hydroxylase is identical to factor inhibiting HIF (FIH) and is related to the cupin structural family. J. Biol. Chem..

[9] Cutter AR, Hayes JJ (2015). A brief review of nucleosome structure. FEBS Lett..

[10] Venkatesh S, Workman JL (2015). Histone exchange, chromatin structure and the regulation of transcription. Nat. Rev. Mol. Cell Biol..

[11] Clapier CR, Cairns BR (2009). The biology of chromatin remodeling complexes. Annu. Rev. Biochem..

[12] Bannister AJ, Kouzarides T (2011). Regulation of chromatin by histone modifications. Cell Res..

[13] Yi SJ, Kim K (2018). Histone tail cleavage as a novel epigenetic regulatory mechanism for gene expression. BMB Rep..

[14] Albanese A, Daly LA, Mennerich D, Kietzmann T, See V (2020). The role of hypoxia-inducible factor post-translational modifications in regulating its localisation, stability, and activity. Int. J. Mol. Sci..

[15] Yi SJ (2019). Bone remodeling: histone modifications as fate determinants of bone cell differentiation. Int. J. Mol. Sci..

[16] Hyun K, Jeon J, Park K, Kim J (2017). Writing, erasing and reading histone lysine methylations. Exp. Mol. Med..

[17] Yang Q (2018). Epigenetics in ovarian cancer: premise, properties, and perspectives. Mol. Cancer.

[18] D’Oto A, Tian QW, Davidoff AM, Yang J (2016). Histone demethylases and their roles in cancer epigenetics. J. Med. Oncol. Ther..

[19] Herz HM, Garruss A, Shilatifard A (2013). SET for life: biochemical activities and biological functions of SET domain-containing proteins. Trends Biochem. Sci..

[20] Chopra A, Cho WC, Willmore WG, Biggar KK (2020). Hypoxia-inducible lysine methyltransferases: G9a and GLP hypoxic regulation, non-histone substrate modification, and pathological relevance. Front. Genet..

[21] Lee JS (2010). Negative regulation of hypoxic responses via induced Reptin methylation. Mol. Cell.

[22] Lee JS (2011). Hypoxia-induced methylation of a pontin chromatin remodeling factor. Proc. Natl Acad. Sci. USA.

[23] Bao L (2018). Methylation of hypoxia-inducible factor (HIF)-1alpha by G9a/GLP inhibits HIF-1 transcriptional activity and cell migration. Nucleic Acids Res..

[24] Casciello F (2017). G9a drives hypoxia-mediated gene repression for breast cancer cell survival and tumorigenesis. Proc. Natl Acad. Sci. USA.

[25] Heddleston JM (2012). Hypoxia-induced mixed-lineage leukemia 1 regulates glioma stem cell tumorigenic potential. Cell Death Differ..

[26] Ortmann BM (2021). The HIF complex recruits the histone methyltransferase SET1B to activate specific hypoxia-inducible genes. Nat. Genet..

[27] Chen S (2016). Hypoxia induces TWIST-activated epithelial-mesenchymal transition and proliferation of pancreatic cancer cells in vitro and in nude mice. Cancer Lett..

[28] Wang X (2020). A lncRNA coordinates with Ezh2 to inhibit HIF-1alpha transcription and suppress cancer cell adaption to hypoxia. Oncogene.

[29] Liu X (2015). Repression of hypoxia-inducible factor alpha signaling by Set7-mediated methylation. Nucleic Acids Res..

[30] Cao L (2020). Downregulation of SETD7 promotes migration and invasion of lung cancer cells via JAK2/STAT3 pathway. Int. J. Mol. Med..

[31] Kim Y (2016). Methylation-dependent regulation of HIF-1alpha stability restricts retinal and tumour angiogenesis. Nat. Commun..

[32] Cohn O, Feldman M, Weil L, Kublanovsky M, Levy D (2016). Chromatin associated SETD3 negatively regulates VEGF expression. Sci. Rep..

[33] Jiang X, Li T, Sun J, Liu J, Wu H (2018). SETD3 negatively regulates VEGF expression during hypoxic pulmonary hypertension in rats. Hypertens. Res..

[34] Lee JY (2017). LSD1 demethylates HIF1alpha to inhibit hydroxylation and ubiquitin-mediated degradation in tumor angiogenesis. Oncogene.

[35] Sacca CD (2019). Inhibition of lysine-specific demethylase LSD1 induces senescence in Glioblastoma cells through a HIF-1alpha-dependent pathway. Biochim. Biophys. Acta Gene Regul. Mech..

[36] Kim I, Park JW (2020). Hypoxia-driven epigenetic regulation in cancer progression: a focus on histone methylation and its modifying enzymes. Cancer Lett..

[37] Beyer S, Kristensen MM, Jensen KS, Johansen JV, Staller P (2008). The histone demethylases JMJD1A and JMJD2B are transcriptional targets of hypoxia-inducible factor HIF. J. Biol. Chem..

[38] Wellmann S (2008). Hypoxia upregulates the histone demethylase JMJD1A via HIF-1. Biochem. Biophys. Res. Commun..

[39] Tausendschon M, Dehne N, Brune B (2011). Hypoxia causes epigenetic gene regulation in macrophages by attenuating Jumonji histone demethylase activity. Cytokine.

[40] Lee HY, Yang EG, Park H (2013). Hypoxia enhances the expression of prostate-specific antigen by modifying the quantity and catalytic activity of Jumonji C domain-containing histone demethylases. Carcinogenesis.

[41] Mimura I (2012). Dynamic change of chromatin conformation in response to hypoxia enhances the expression of GLUT3 (SLC2A3) by cooperative interaction of hypoxia-inducible factor 1 and KDM3A. Mol. Cell. Biol..

[42] Dobrynin G (2017). KDM4A regulates HIF-1 levels through H3K9me3. Sci. Rep..

[43] Hancock RL, Masson N, Dunne K, Flashman E, Kawamura A (2017). The activity of JmjC histone lysine demethylase KDM4A is highly sensitive to oxygen concentrations. ACS Chem. Biol..

[44] Fu L (2012). HIF-1alpha-induced histone demethylase JMJD2B contributes to the malignant phenotype of colorectal cancer cells via an epigenetic mechanism. Carcinogenesis.

[45] Luo W, Chang R, Zhong J, Pandey A, Semenza GL (2012). Histone demethylase JMJD2C is a coactivator for hypoxia-inducible factor 1 that is required for breast cancer progression. Proc. Natl Acad. Sci. USA.

[46] Gallipoli P, Huntly BJP (2019). Histone modifiers are oxygen sensors. Science.

[47] Batie M (2019). Hypoxia induces rapid changes to histone methylation and reprograms chromatin. Science.

[48] Zhou X (2010). Hypoxia induces trimethylated H3 lysine 4 by inhibition of JARID1A demethylase. Cancer Res..

[49] Chakraborty AA (2019). Histone demethylase KDM6A directly senses oxygen to control chromatin and cell fate. Science.

[50] Li Y (2019). Kdm6A protects against hypoxia-induced cardiomyocyte apoptosis via H3K27me3 demethylation of Ncx gene. J. Cardiovasc. Transl. Res..

[51] Liu OH (2020). Hypoxia-mediated regulation of histone demethylases affects angiogenesis-associated functions in endothelial cells. Arterioscler. Thromb. Vasc. Biol..

[52] Prickaerts P (2016). Hypoxia increases genome-wide bivalent epigenetic marking by specific gain of H3K27me3. Epigenetics Chromatin.

[53] Schneider A (2013). Acetyltransferases (HATs) as targets for neurological therapeutics. Neurotherapeutics.

[54] Park SY, Kim JS (2020). A short guide to histone deacetylases including recent progress on class II enzymes. Exp. Mol. Med..

[55] Li G, Tian Y, Zhu WG (2020). The roles of histone deacetylases and their inhibitors in cancer therapy. Front. Cell Dev. Biol..

[56] Arany Z (1996). An essential role for p300/CBP in the cellular response to hypoxia. Proc. Natl Acad. Sci. USA.

[57] Chen R (2012). The acetylase/deacetylase couple CREB-binding protein/Sirtuin 1 controls hypoxia-inducible factor 2 signaling. J. Biol. Chem..

[58] Ruas JL (2010). Complex regulation of the transactivation function of hypoxia-inducible factor-1 alpha by direct interaction with two distinct domains of the CREB-binding protein/p300. J. Biol. Chem..

[59] Geng H (2012). HIF1alpha protein stability is increased by acetylation at lysine 709. J. Biol. Chem..

[60] Yin S (2012). Arylsulfonamide KCN1 inhibits in vivo glioma growth and interferes with HIF signaling by disrupting HIF-1alpha interaction with cofactors p300/CBP. Clin. Cancer Res.

[61] Lim JH (2010). Sirtuin 1 modulates cellular responses to hypoxia by deacetylating hypoxia-inducible factor 1alpha. Mol. Cell.

[62] Feng X (2021). Hypoxia-induced acetylation of PAK1 enhances autophagy and promotes brain tumorigenesis via phosphorylating ATG5. Autophagy.

[63] Perez-Perri JI (2016). The TIP60 complex is a conserved coactivator of HIF1A. Cell Rep..

[64] Wang M (2021). Lack of MOF decreases susceptibility to hypoxia and promotes multidrug resistance in hepatocellular carcinoma via HIF-1alpha. Front. Cell Dev. Biol..

[65] Kim MS (2001). Histone deacetylases induce angiogenesis by negative regulation of tumor suppressor genes. Nat. Med..

[66] Liang D, Kong X, Sang N (2006). Effects of histone deacetylase inhibitors on HIF-1. Cell Cycle.

[67] Fath DM (2006). Histone deacetylase inhibitors repress the transactivation potential of hypoxia-inducible factors independently of direct acetylation of HIF-alpha. J. Biol. Chem..

[68] Jeon HW, Lee YM (2010). Inhibition of histone deacetylase attenuates hypoxia-induced migration and invasion of cancer cells via the restoration of RECK expression. Mol. Cancer Ther..

[69] Liu M (2019). Hypoxia-induced feedback of HIF-1alpha and lncRNA-CF129 contributes to pancreatic cancer progression through stabilization of p53 protein. Theranostics.

[70] Zhao B (2020). HIF-1alpha and HDAC1 mediated regulation of FAM99A-miR92a signaling contributes to hypoxia induced HCC metastasis. Signal Transduct. Target. Ther..

[71] Pluemsampant S, Safronova OS, Nakahama K, Morita I (2008). Protein kinase CK2 is a key activator of histone deacetylase in hypoxia-associated tumors. Int. J. Cancer.

[72] Chang CC (2011). HDAC2 promotes cell migration/invasion abilities through HIF-1alpha stabilization in human oral squamous cell carcinoma. J. Oral. Pathol. Med..

[73] Wu MZ (2011). Interplay between HDAC3 and WDR5 is essential for hypoxia-induced epithelial-mesenchymal transition. Mol. Cell.

[74] Safronova OS, Nakahama K, Morita I (2014). Acute hypoxia affects P-TEFb through HDAC3 and HEXIM1-dependent mechanism to promote gene-specific transcriptional repression. Nucleic Acids Res..

[75] Wang J (2021). Repression of the miR-627-5p by histone deacetylase 3 contributes to hypoxia-induced hepatocellular carcinoma progression. J. Cancer.

[76] Qian DZ (2006). Class II histone deacetylases are associated with VHL-independent regulation of hypoxia-inducible factor 1α. Cancer Res..

[77] Geng H (2011). HDAC4 protein regulates HIF1alpha protein lysine acetylation and cancer cell response to hypoxia. J. Biol. Chem..

[78] Seo HW, Kim EJ, Na H, Lee MO (2009). Transcriptional activation of hypoxia-inducible factor-1alpha by HDAC4 and HDAC5 involves differential recruitment of p300 and FIH-1. FEBS Lett..

[79] Chen S (2015). AMPK-HDAC5 pathway facilitates nuclear accumulation of HIF-1alpha and functional activation of HIF-1 by deacetylating Hsp70 in the cytosol. Cell Cycle.

[80] Kong X (2006). Histone deacetylase inhibitors induce VHL and ubiquitin-independent proteasomal degradation of hypoxia-inducible factor 1alpha. Mol. Cell. Biol..

[81] Ryu HW, Won HR, Lee DH, Kwon SH (2017). HDAC6 regulates sensitivity to cell death in response to stress and post-stress recovery. Cell Stress Chaperones.

[82] Zhang Z (2019). Increase in HDAC9 suppresses myoblast differentiation via epigenetic regulation of autophagy in hypoxia. Cell Death Dis..

[83] Yoon H, Shin SH, Shin DH, Chun YS, Park JW (2014). Differential roles of Sirt1 in HIF-1alpha and HIF-2alpha mediated hypoxic responses. Biochem. Biophys. Res. Commun..

[84] Ryu DR (2019). Sirt1-hypoxia-inducible factor-1alpha interaction is a key mediator of tubulointerstitial damage in the aged kidney. Aging Cell.

[85] Dioum EM (2009). Regulation of hypoxia-inducible factor 2alpha signaling by the stress-responsive deacetylase sirtuin 1. Science.

[86] Seo KS (2015). SIRT2 regulates tumour hypoxia response by promoting HIF-1alpha hydroxylation. Oncogene.

[87] Kaitsuka T, Matsushita M, Matsushita N (2020). SIRT2 inhibition activates hypoxia-inducible factor 1alpha signaling and mediates neuronal survival. Biochem. Biophys. Res. Commun..

[88] Tseng AH, Wu LH, Shieh SS, Wang DL (2014). SIRT3 interactions with FOXO3 acetylation, phosphorylation and ubiquitinylation mediate endothelial cell responses to hypoxia. Biochem. J..

[89] Yang Z, Yu W, Huang R, Ye M, Min Z (2019). SIRT6/HIF-1alpha axis promotes papillary thyroid cancer progression by inducing epithelial-mesenchymal transition. Cancer Cell Int..

[90] Yang Z (2021). SIRT6 promotes angiogenesis and hemorrhage of carotid plaque via regulating HIF-1alpha and reactive oxygen species. Cell Death Dis..

[91] Hubbi ME, Hu H, Kshitiz, Gilkes DM, Semenza GL (2013). Sirtuin-7 inhibits the activity of hypoxia-inducible factors. J. Biol. Chem..

[92] Adriaens ME (2016). Quantitative analysis of ChIP-seq data uncovers dynamic and sustained H3K4me3 and H3K27me3 modulation in cancer cells under hypoxia. Epigenetics Chromatin.

[93] Lee S (2017). Multi-dimensional histone methylations for coordinated regulation of gene expression under hypoxia. Nucleic Acids Res..

[94] Batie M, Frost J, Shakir D, Rocha S (2022). Regulation of chromatin accessibility by hypoxia and HIF. Biochem. J..

[95] Wang J, Wang Y, Duan Z, Hu W (2020). Hypoxia-induced alterations of transcriptome and chromatin accessibility in HL-1 cells. IUBMB Life.

[96] Li Y (2020). Acetate supplementation restores chromatin accessibility and promotes tumor cell differentiation under hypoxia. Cell Death Dis..

[97] Ward MC, Banovich NE, Sarkar A, Stephens M, Gilad Y (2021). Dynamic effects of genetic variation on gene expression revealed following hypoxic stress in cardiomyocytes. Elife.

[98] Xin J (2020). Chromatin accessibility landscape and regulatory network of high-altitude hypoxia adaptation. Nat. Commun..

[99] Chen PS (2020). Pathophysiological implications of hypoxia in human diseases. J. Biomed. Sci..

[100] Lee JW, Ko J, Ju C, Eltzschig HK (2019). Hypoxia signaling in human diseases and therapeutic targets. Exp. Mol. Med..

[101] Harris AL (2002). Hypoxia-a key regulatory factor in tumour growth. Nat. Rev. Cancer.

[102] Kang HS (2020). Intermittent hypoxia exacerbates tumor progression in a mouse model of lung cancer. Sci. Rep..

[103] Tanaka S, Tanaka T, Nangaku M (2016). Hypoxia and hypoxia-inducible factors in chronic kidney disease. Ren. Replacement Ther..

[104] Tanemoto F, Mimura I (2022). Therapies targeting epigenetic alterations in acute kidney injury-to-chronic kidney disease transition. Pharmaceuticals (Basel).

[105] Funk JA, Schnellmann RG (2012). Persistent disruption of mitochondrial homeostasis after acute kidney injury. Am. J. Physiol. Ren. Physiol..

[106] Ha SD, Solomon O, Akbari M, Sener A, Kim SO (2018). Histone deacetylase 8 protects human proximal tubular epithelial cells from hypoxia-mimetic cobalt- and hypoxia/reoxygenation-induced mitochondrial fission and cytotoxicity. Sci. Rep..

[107] Liu M (2020). Novel therapeutic targets for hypoxia-related cardiovascular diseases: the role of HIF-1. Front. Physiol..

[108] Abe H, Semba H, Takeda N (2017). The roles of hypoxia signaling in the pathogenesis of cardiovascular diseases. J. Atheroscler. Thromb..

[109] Gupta, N.A. & Ashraf, M. Z. (eds) *Hypoxia Signaling in Cardiovascular Diseases* (IntechOpen, London, 2018).

[110] Zhang W, Song M, Qu J, Liu GH (2018). Epigenetic modifications in cardiovascular aging and diseases. Circ. Res..

[111] Bustelo M (2020). Clinical implications of epigenetic dysregulation in perinatal hypoxic-ischemic brain damage. Front. Neurol..

[112] Xue H (2019). Sevoflurane post-conditioning alleviates neonatal rat hypoxic-ischemic cerebral injury via Ezh2-regulated autophagy. Drug Des. Devel. Ther..

[113] Cox-Limpens KE, Vles JSDLAVDH, Zimmermann LJ, Gavilanes AW (2014). Fetal asphyctic preconditioning alters the transcriptional response to perinatal asphyxia. BMC Neurosci..

[114] Cox-Limpens KE (2013). Fetal brain genomic reprogramming following asphyctic preconditioning. BMC Neurosci..

[115] Ziemka-Nalecz M (2017). Sodium butyrate, a histone deacetylase inhibitor, exhibits neuroprotective/neurogenic effects in a rat model of neonatal hypoxia-ischemia. Mol. Neurobiol..

[116] Kabakus N (2005). Protective effects of valproic acid against hypoxic-ischemic brain injury in neonatal rats. J. Child Neurol..

[117] Faraco G (2006). Pharmacological inhibition of histone deacetylases by suberoylanilide hydroxamic acid specifically alters gene expression and reduces ischemic injury in the mouse brain. Mol. Pharmacol..

[118] Kim HJ (2007). Histone deacetylase inhibitors exhibit anti-inflammatory and neuroprotective effects in a rat permanent ischemic model of stroke: multiple mechanisms of action. J. Pharmacol. Exp. Ther..

[119] Jaworska J, Zalewska T, Sypecka J, Ziemka-Nalecz M (2019). Effect of the HDAC inhibitor, sodium butyrate, on neurogenesis in a rat model of neonatal hypoxia-ischemia: potential mechanism of action. Mol. Neurobiol..

[120] Semenza GL (2012). Hypoxia-inducible factors in physiology and medicine. Cell.

[121] Prabhakar NR, Peng YJ, Nanduri J (2020). Hypoxia-inducible factors and obstructive sleep apnea. J. Clin. Invest..

[122] Wang N, Peng YJ, Su X, Prabhakar NR, Nanduri J (2021). Histone deacetylase 5 is an early epigenetic regulator of intermittent hypoxia induced sympathetic nerve activation and blood pressure. Front. Physiol..

[123] Martinez, C. A., Bal, N., Cistulli, P. A. & Cook, K. M. Intermittent hypoxia enhances the expression of HIF1A by increasing the quantity and catalytic activity of KDM4A-C and demethylating H3K9me3 at the HIF1A locus. Preprint at https://biorxiv.org/content/10.1101/2021.07.25.453726v1 (2021).

[124] Qian DZ (2006). Class II histone deacetylases are associated with VHL-independent regulation of hypoxia-inducible factor 1 alpha. Cancer Res..

